# Risk Factors for Hearing Loss and Its Prevalence in Neonates Older than 6 Months with History of Hospitalization in Intensive Care Unit

**Published:** 2018

**Authors:** Zarrin KEIHANIDOST, Aydin TABRIZI, Elahe AMINI, Mojtaba SEDAGHAT, AmirAli GHAHREMANI, Mamak SHARIAT, Zeinab KAVYANI

**Affiliations:** 1 Department of Pediatrics, School of Medicine, Tehran University of Medical Science, Tehran, Iran; 2 Pediatric Neurology Research Center, Shahid Beheshti University of Medical Siences, Tehran, Iran.; 3 Department of Neurology, School of Medicine, Mashhad University of Medical Science, Mashhad, Iran; 4 Maternal-Fatal and Neonatal Research Center, Tehran University of Medical Science, Tehran, Iran

**Keywords:** Hearing loss, Neonates, Risk factor, Auditory Brainstem Response

## Abstract

**Objectives:**

Hearing loss is one of the most important disabilities in neonates. Delay in the detection of hearing loss leads to impaired development and may prevent the acquisition of speech. We aimed to determine the risk factors associated with hearing loss in neonatal patients aged more than 6 months with a history of hospitalization in Neonatal Intensive Care Unit (NICU).

**Methods:**

In this case-control study, screening for hearing loss was carried out on 325 neonates aged 6-12 months referred to Pediatric Neurology Office of Vali-e-Asr Hospital, Tehran, Iran up to 2011. Hearing loss was confirmed using Auditory Brainstem Response screening test (ABR).

**Results:**

The prevalence of mildly and moderately hearing loss in neonates was determined as 3.6%. The most significant risk factors for hearing loss in neonates were neonatal icterus associated with phototherapy, respiratory distress syndrome (RDS) and lower Apgar score.

**Conclusion:**

It seems to quantitative auditory system screening using ABR is necessary for all neonates; because rehabilitation support such as speech therapy and hearing training in this age period is more effective than older ages.

## Introduction

One of the valuable human senses is hearing that facilitate communication between people through speaking (1). Hearing loss in early life lays a disability that prevents the evolution of speech skill, also language and hearing difficulties (2, 3). Neonatal hearing loss is estimated at approximately 1 to 3 cases per 100 live births increases to 1%-5% in infants hospitalized in neonatal intensive care units (NICU) (4). The severity of hearing loss defines based on the assessment of threshold in decibels (dB) at various frequencies; normal hearing threshold is 0-20 dB and the severity of hearing loss ranks to 4 categories of mild, moderate, severe and profound loss (5). 

The majority of neonatal hearing losses are sensorineural (6), but according to Joint Committee on Infant Hearing (JCIH), neonatal risk factors are risk factors for neonatal hearing loss as follows:

a) family history of hearing impairment, b)intrauterine infections such as cytomegalovirus (CMV), toxoplasma etc., c) neonatal specific disorders as severe hyperbilirubinemia that needs exchange transfusion, d) persistence pulmonary hypertension (PPHN) associated with mechanical ventilation and e) after birth infection like bacterial meningitis (7). By 1998, screening for hearing loss was done in 2.5 years of age in the United States. By implementing public hearing screening program in 2001, the average age of diagnosis was commuted to 3 months and the average age of the intervention determined as 6 months (8).

According to American Pediatrics Academy (APA), the effective test for neonatal hearing screening should detect hearing loss more than 30 dB and be accurate in lower than 3-month neonates; two electrophysiological tests have these properties: Auditory Brainstem Responses (ABR) and Otoacoustic Emission (OAE) (9). ABR measures action potentials from cranial nerve of eight (cochlear nerve) to lower midbrain colliculus in response to the incoming stimulus (3). ABR measures not only the integrity of the inner ear but also the auditory pathway (10). ABR responses are less subjective than common behavioral tests and also can assess mild or unilateral hearing loss. Validity, reliability and predictive efficiency of ABR in neonatal hearing loss detection have been proven already (11, 12).

"Hearing loss causes delays in development of speech and language, and those delays then lead to learning problems, often resulting in poor school performance" (8). Nowadays, in the health centers in Iran, hearing status assessment of newborns is done with OAE qualitative test and ABR in 6-12 month neonates do not perform routinely.

We aimed to evaluate hearing loss in 6-12 months of neonates with a history of NICU admission.

## Materials & Methods

This case-control study was conducted for further investigations of hearing loss in 6-12 months neonates referred to Pediatric Neurology Clinic (Tehran, Iran) for 5 years up to August 2011, with a history of hospitalization in Vali-e-Asr Hospital's NICU, Tehran, Iran. 

Overall, 325 neonates were enrolled based on the inclusion criteria. Infants clinically and para clinically proved hearing loss assumed as case group and the control group was those who had no hearing impairment. An auditory evoked potential system (Charter ICS, Denmark) was used to record ABR. A click-type stimulus was used twice with rarefaction polarity, with Intensity of 35-80 dB NHL, under 21/1 pulse set based on 1500 trials and analysis time of 10-15 ms. All of the neonates had been sedated by oral chlorate hydrate, 50 mg/kg half an hour before the test. 

Four electrodes were applied as follows: two active ones were placed on mastoid bones, the reference electrode on vertex, and the ground electrode on the forehead. Recorded waves were analyzed and interpreted by an expert audiologist. Measurable and comparable variables consisted of mean latency of V, III and I waves, interpeak interval of I-III, III-V, I-V waves and no waves. Neonates were divided into two groups according to the normal and abnormal results of the ABR. After assessment of hearing status, 12 neonates showed impairment (case group) and 4 times more neonates enrolled as a control group (n=48). Both of groups matched in gestational age (± 2 wk) and birth weight (± 200 gr). Hearing loss detected using ABR test. Recorded variables collected in 3 categories:

1. Pregnancy variables: A) fetal infections e.g. CMV, rubella, hepatitis B, HIV, syphilis, and chickenpox; B) mothers drug abuse during pregnancy; C) mothers diseases including preeclampsia, gestational diabetes mellitus (GDM), chronic diabetes mellitus, chronic hypertension and heart diseases.

2. Neonatal variables: mechanical ventilation more than 2 d, low Apgar score (<7), RDS, metabolic diseases (including congenital adrenal hyperplasia, galactosemia, Phenylketonuria (PKU) and hyper/hypothyroidism), sepsis, icterus requiring phototherapy or exchange transfusion.

3. Independent variables: hearing loss of mothers, fathers or both of them.

Informed consent was received verbally from parents. The name and personal information of the patients remained confidential 

Statistical Analysis

Data were analyzed using SPSS software 17.2 (Chicago, IL, USA). Bijective dependent variables analyzed via logistic regression; student t-test used for assessment of correlations among quantitative variables including Apgar score, mechanical ventilation days and qualitative variables including history of icterus, RDS, PPROM sepsis, preeclampsia, thyroid dysfunction and Delivery type analyzed by Chi-square test. *A P*-value of less than 0.05 considered as significant and for significant correlations, Odd ration with confidence interval of 95% calculated for significant correlations.

## Results

Among 325 neonates, 12 infants (3.6%) showed hearing loss according to ABR results as mild (threshold of hearing 15-30 db) and moderate (30-50 db) hearing loss. There was 3 females (25%) and 9 males (75%) in these 12 cases with male to female ratio as 3 to 1. Bilateral hearing loss was shown in 3 neonates (0.92%).

History of icterus, acute RDS and low Apgar score showed significant with impaired hearing screening results (Table 1).

**Table 1:** **Correlation of impaired hearing screening results with History of icterus, acute RDS and low Apgar score**

Variables		Percentage	Correlation with Hearing Loss		
			OR	CI 95%	*P*-value
Icterus	Case	75	4.5	2.2-9.1	<0.001
	Control	16.7			
RDS	Case	25	0.75	0.54-1	0.006
	Control	0			
Low Apgar score	Case	33.3	5.3	1.3-20.7	0.02
	Control	6.3			

There were no histories of meconium-stained liquor, maternal heart diseases and diabetes in both groups. In the history of any of the members of the two groups, there was no evidence of TORCH. The family history of hearing impairment was negative for members of both groups. Number and percent of not significant variables are listed in Table 2.

**Table 2:** **Number and percent of not significant variables**

			Number of cases	Percent
Delivery type	NVD	Case	5	41.7
		Control	12	25
	CS	Case	7	58.3
		Control	36	75
Sepsis	Case		1	8.3
	Control		5	10.4
PROM	Case		3	25
	Control		4	8.3
Mothers preeclampsia	Case		2	16.7
	Control		1	2.1
Thyroid dysfunction	Case		0	0
	Control		1	2.1
Mechanical ventilation ≥2 d	Case		1	8.3
	Control		1	2.1

No significant differences were noticed between case and control groups in Delivery type, Sepsis, PROM, preeclampsia, thyroid dysfunction and ventilation; NVD: normal vaginal delivery, CS: cesarean section, PROM: preterm rupture of membrane.

## Discussion

In the present study, 3.6% of infants showed hearing loss according to ABR result with male to female ratio as 3 to 1. All of infants in case group showed impaired OAE test and 75% of these infants had a history of icterus while only 16.7% of infants in control group had this history. A history of acute RDS showed in 25% of neonates in case group and there was no case of RDS in control group. Low Apgar score was recorded for 33.3% of neonates in case group and 6.3% of cases in control group. 

The total cost of education for not screened hard-hearing children imposes high costs on governments (13). The prevalence of congenital hearing impairment determines in neonatal screening, was 2 times more than other disorders characterized by the screening of this age period (14, 15). The frequency of this disorder was 2-6 cases per 100 live births, the figures obtained similar results in Iran which implies that the hearing loss is a health problem in our country (6, 15). In a pilot study of national hearing screening in Iran, 89.7% of newborns proved normal in the primary screening in the maternity ward using OAE test, and 10.3% were abnormal (13). Most of the infants born in hospitals of the project were not referred for follow-up process of hearing screening. Using Poison's distribution for frequency rate of hearing impairment and normal estimate for this distribution and 95% confidence intervals, significant bilateral sensorineural hearing loss was present in ~1 to 4 per 1000 live births in the well-baby nursery population (12 neonates), and in ~2.5 to 4.6 per 100 infants in the intensive care unit (13). The reasons for this situation, especially in Iran are cultural, economic and family health and community awareness of the adverse consequences of hearing impairment in newborns that are discussable from different perspectives. 

Our results showed that the prevalence of hearing loss in infants with risk factors before and after birth was 3.6 cases per 100 live births; this figure was almost within the global prevalence (7). In Iran, this prevalence was 8(16), 3.5 (8) and 28 (17) cases per 100 births. Several reports from different regions of the world expressed wide ranges of hearing loss prevalence as 7.8%, 29.1% and 13.5% (18-20). The reason for this variation in results may be using different protocols for screening and also real difference in the prevalence of the disorder in different regions of the world. In a 4 year period, evaluate hearing status 15165 newborns at 15^th^ d after birth using OAE test and the prevalence of hearing impairment showed in 10.8% cases in this stage; ABR test was done for these neonates in next stage and hearing loss confirmed for 6.2% by ABR (10). This study showed that routine screen in the health centers in Iran overestimates hearing loss; so, OAE should be replaced with a more accurate test in early stage. 

A research on 1062 infants at risk for hearing loss showed 14 patients with bilateral hearing loss (21). The incidence of bilateral hearing loss was reported as 1-4/1000 newborns (22); however present results showed bilateral hearing loss in 0.9% of infants. Differences in outcome may depend on the type of hospital and its patients. The current study hospital is one of the reference hospitals for women with high-risk pregnancies and infants which may result in higher percentage of neonatal hearing loss compared to similar studies in Iran.

An important risk factor for hearing impairment in this study was icterus requiring phototherapy (OR=4.5). Nowadays, the importance of icterus, especially severe degrees, is well marked in auditory neuropathy (17, 23). The frequency of hearing impairment in neonates born by normal vaginal delivery (NVD) was significantly more than cesarean section (13). However, in the present results, this correlation was not seen; probably due to low sample size and professionally done NVD in our study population. Low birth Apgar score was significantly correlated with subsequent hearing loss (OR= 5.3), reported as a similar rate already (24, 25); however, this connection was not proven yet (3).

The present study showed that there was no correlation between maternal preeclampsia and neonatal hearing loss, reported already (26). Neonates with hyperbilirubinemia history show higher frequency of hearing loss up to 5 times more than neonates without icterus (27). This relationship also was noticed in our study where 75% of icteric neonates were found with hearing impairment. Low Apgar score was found as a risk factor for hearing loss (28); in line with our results that neonates with low Apgar score showed following hearing loss about 5 times more than normal neonates. There was no correlation between neonatal hypothyroidism and hearing loss (29). Our result showed similar finding on thyroid dysfunction including hyper/hypothyroidism are not related to hearing loss.

The present findings showed correlation between neonatal RDS and hearing loss; however few studies investigate this relationship. RDS was not a risk factor for hearing loss (18); while a case of 2 infants with RDS showed delayed hearing loss at the age of 2.5 yr; who had normal hearing status up to 1-year screening programs (30). These controversial results should be clear by further evaluations.

In conclusion, the newborn hearing screening program is possible, beneficial and justified in terms of scientific and economic principles that provide early treatment interventions that can significantly reduce further costs for patients. Moreover, because the incidence of congenital bilateral hearing loss is higher than the prevalence of other disorder screening, newborn hearing screening is done as soon as possible-preferably before three months of age and early intervention programs and the hearing strengthening tools should be available in health centers. Given the prevalence of hearing impairment 3.6% and risk factors such as phototherapy treated icterus, RDS and low birth Apgar score and also the cost of hard-hearing patients training, it seems necessary considering of these risk factors in hearing screening. Nowadays, neonatal hearing status screening performs routinely using OAE in Iran; however, ABR is a more accurate test to replace with OAE in the first step of screening.
